# Detection Method of Stator Coating Quality of Flat Wire Motor Based on Improved YOLOv8s

**DOI:** 10.3390/s24165392

**Published:** 2024-08-21

**Authors:** Hongping Wang, Gong Chen, Xin Rong, Yiwen Zhang, Linsen Song, Xiao Shang

**Affiliations:** 1 School of Mechanical and Electrical Engineering, Changchun University of Science and Technology, Changchun 130012, China; 2 Faw Tooling Die Manufacturing Co., Ltd., Lvyuan, Changchun 130013, China

**Keywords:** tiny defect detection, YOLO, stators of flat wire motor, DSFI-HEAD, LEFG

## Abstract

The stator of a flat wire motor is the core component of new energy vehicles. However, detecting quality defects in the coating process in real-time is a challenge. Moreover, the number of defects is large, and the pixels of a single defect are very few, which make it difficult to distinguish the defect features and make accurate detection more difficult. To solve this problem, this article proposes the YOLOv8s-DFJA network. The network is based on YOLOv8s, which uses DSFI-HEAD to replace the original detection head, realizing task alignment. It enhances joint features between the classification task and localization task and improves the ability of network detection. The LEFG module replaces the C2f module in the backbone of the YOLOv8s network that reduces the redundant parameters brought by the traditional BottleNeck structure. It also enhances the feature extraction and gradient flow ability to achieve the lightweight of the network. For this research, we produced our own dataset of stator coating quality regarding flat wire motors. Data augmentation technology (Gaussian noise, adjusting brightness, etc.) enriches the dataset, to a certain extent, which improves the robustness and generalization ability of YOLOv8s-DFJA. The experimental results show that in the performance of YOLOv8s-DFJA compared with YOLOv8s, the mAP@.5 index increased by 6.4%, the precision index increased by 1.1%, the recall index increased by 8.1%, the FPS index increased by 9.8FPS/s, and the parameters decreased by 3 Mb. Therefore, YOLOv8s-DFJA can be better realize the fast and accurate detection of the stator coating quality of flat wire motors.

## 1. Introduction

**Figure 1:**
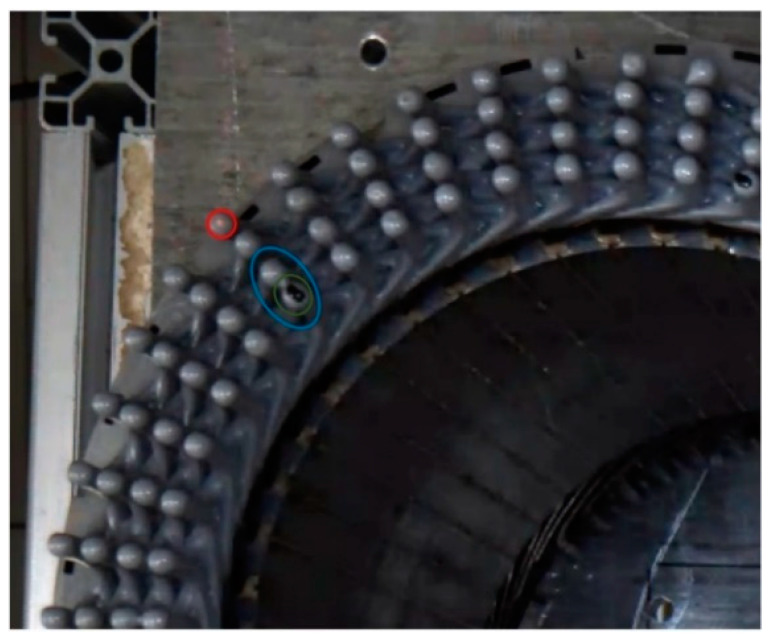
Image samples of stator coating area defects. The red ring is a bare defect, the blue ring is an adhesion defect, and the green ring is an impurity defect.

With the continuous development of science and technology and the improvement of quality of life, people pay more and more attention to environmental protection [1]. Compared with the greenhouse gas produced by oil vehicles, the importance of clean and environmental protection of new energy vehicles is increasing day by day. As the core component of new energy vehicles, the stator of a flat wire motor is popular in the new energy vehicle industry because of its advantages, including an about 30% higher slot filling rate [2] than the traditional round-wire winding with bare-welding parts. To ensure the safe, efficient, and long-term use of the motor, it is necessary to coat the flat wire of the flat wire motor stator with high-reliability insulation [3]. In the flat wire motor stator manufacturing process, the solder joint area should be coated with epoxy resin after laser welding. There are three main defects in the stator of the flat wire motor with unqualified coating quality: copper leakage defect, adhesion defect, and impurity defect, as shown in Figure 1. It is necessary to find and deal with the defects of the stator of the flat wire motor in the manufacturing process in time to avoid potential safety hazards. Therefore, it is necessary to accurately detect the coating quality of the stator of the flat wire motor to ensure its safety, stability, and reliability during operation so as to prevent failure.

The automatic production line of flat wire motors has high production capacity and efficiency requirements, generally reaching 60 s/pcs to 150 s/pcs. Therefore, the detection speed is also crucial. The traditional manual detection method has the disadvantages of poor real-time performance, low detection accuracy, and poor environmental adaptability, which makes it difficult to meet the requirements of real-time and accurate detection of industrial surface defects. If one wants to achieve the purpose of timely error correction’ one must analyze the image in real time and provide the test results so as to minimize the number of defective products, reduce waste, and overall production costs [4]. In recent years, surface defect detection technology based on machine vision has gradually replaced manual detection [5]. This has the advantages of high precision, high efficiency, and non-contact measurement and is widely used in the detection of various workpiece surfaces [6], such as flat steel [7], textiles [8], mobile phone screens [9], fruits/vegetables [10], rails [11], leather [12], battery materials [13], and other fields. Visual detection methods are usually divided into traditional image processing and deep learning. Both methods should consider three indicators: accuracy, robustness, and speed. Traditional image processing detection methods can be divided into statistical methods, spectral methods, network-based methods, and learning-based methods [14]. They usually need to be designed according to the specific operation of the image and first converted into digital images to highlight the relevant features, followed by feature extraction of ROI (Region of Interest); finally, the automatic classifier is used to explain the content of the image. These tedious operations not only require experienced visual engineers to complete them manually but also need to be fine-tuned for specific problems. In different environments, they do not have adaptability. To a certain extent, this reflects the shortcomings of traditional image processing methods, such as poor generalization ability and harsh application conditions. In recent years, deep learning has shown superior performance in industrial defect detection. Still, there are also some shortcomings, for example, the dataset of workpiece surface defects is usually tiny and difficult to collect or mark, and the training time is extended. Nevertheless, deep learning can autonomously learn advanced features from training data compared to traditional image processing methods and has a more robust generalization performance. For example, Luo et al. [15] proposed an enhanced Mask R-CNN algorithm, which improves the speed and accuracy of insulator defect detection by incorporating the fusion factor into FPN. Wang et al. [16] proposed a method combining improved ResNet50 and Faster R-CNN to reduce the average running time and improve the accuracy of automatic detection and classification of steel surface defects.

Although the convolutional neural network based on Faster R-CNN and Mask R-CNN can be used for accurate detection of surface defects of metal workpieces, its applicability in practical applications is limited due to the long time, slow speed, and low efficiency of the two-stage target detection method. The YOLO network proposed by Redmon et al. [17] is a typical one-stage target detection network that can directly output target candidate boxes and coordinates. With the successive launch of YOLOv2-YOLOv8, more and more researchers use the YOLO series network to complete target recognition and defect detection-related tasks and improve the YOLO network to meet various challenges in detection. For example, Zhang et al. [18] realized the collection and identification of surface cracks on metal pipes by combining annular light and coaxial light and introducing dual attention and boundary refinement modules. Wang et al. [19] adopted the de-weighted BiFPN structure, combined the ECA attention mechanism in the main part, and used the SIoU loss function instead of the original bounding box loss function to achieve efficient detection of strip surface defects. In summary, the existing methods have some shortcomings in real-time accuracy when identifying and locating the solder joint area of the stator of the flat wire motor and detecting defects in the solder joint area. In this scenario, the recognition rate and detection accuracy of some methods cannot meet the requirements, and the reliability of the detection results cannot be ensured. Although other methods have better detection ability, their network complexity is too high, resulting in slow detection speed and enabling guaranteeing real-time performance.

In view of the actual industrial application scenarios, this article designs a DSFI-HEAD detection head and a LEFG lightweight structure. The main contributions are as follows:

- DSFI-HEAD Module: Traditional detection heads often suffer from feature loss or inaccurate localization when dealing with complex backgrounds and small targets. To address this challenge, the DSFI-HEAD module is proposed. This module enhances feature fusion and improves the detection head’s representational capability, thus significantly improving detection accuracy, especially for small targets and in complex scenarios;
- LFEG Module: Conventional algorithms may face issues with increased network complexity and parameter count when handling complex task learning, which can affect the model’s real-time performance and efficiency. To tackle this, the LFEG module is introduced, which reduces network parameters and complexity, optimizing the network structure and enhancing detection speed and efficiency. The core is to use feature extractors to extract and fuse task-related features from multi-layer convolutional networks to generate joint feature maps with richer information. By aligning tasks, the synergy between tasks is improved, thereby improving the accuracy and efficiency of the network.
- Based on YOLOv8s, DSFI-HEAD and LEFG are added to jointly construct the YOLOv8s-DFJA network, which realizes the requirements of network’s lightweightness and real-time accurate detection while ensuring accuracy.

## 2. Related Work

In industrial applications, accurate detection of large objects in images has been achieved, but the accurate detection of small objects is still a challenge [20]. This article focuses on the small defects in the solder joint area of the stator of the flat wire motor. The pixel size of these defects is small, so the image quality requirements are high. Low-quality images make it difficult to distinguish various defect features, the background is complex, and the context clues are limited, which further increases the difficulty of defect detection.

Small-scale changes and huge similarities in complex backgrounds make it difficult to distinguish defects. Some studies have tried to make the network focus on a feature in the image and strengthen the learning of the feature. For example, Zhang et al. [21] reorganized the link between CA attention and SA attention and combined the multi-branch ConvNet structure to improve the detection performance of YOLOv5s for small targets. Zhu et al. [22] replaced the original prediction head of YOLOv5 with a Transformer pre-diction head constructed using the Transformer encoder block and incorporated a self-attention mechanism. This modification enhanced the network’s feature representation capabilities and improved detection accuracy. Kim et al. [23] used an efficient channel attention module to modify the backbone of YOLO and proposed a channel attention pyramid method to improve the ability to detect small targets. Zhang et al. [24] integrated the Contextual Transformer block and attention mechanism into the Darknet-53 backbone, significantly enhancing context information extraction, visual representation of small objects, and the network’s overall expressive ability. Analyzing all of the above research, it was found that while integrating the attention mechanism into the network can enhance its important feature learning ability in the spatial and channel dimensions, this approach typically captures only local information and fails to explore the data’s comprehensiveness fully. 

There are also some studies that try to make the network pay attention to more scale information. For example, Jiang et al. [25] proposed a joint multi-scale defect detection method that constructed a wider and more effective focusing feature to detect small targets accurately. Su et al. [26] enhanced the ability of the YOLO network to detect small defects by multi-scale fusion method of region extraction and 16X down-sampling features and realized the accurate detection of metal gear end-face defects. Wang et al. [27] proposed a method combining multi-scale channel information (MCI) and global local attention (GLA) to enhance the learning ability of the YOLOv7 network and achieve accurate detection of insulator defects. However, the network is too complex, and the parameter amount reaches 40.6 Mb, which cannot meet the real-time detection requirements. The above research shows that some researchers are committed to optimizing the sub-modules of the YOLO network to realize multi-scale feature fusion and enhance its detection ability. However, there are limitations in integrating more extensive context information, especially in small target detection. If the semantic features on a large scale (high level) are too emphasized, it will lead to insufficient capture of detailed information of small targets and vice versa. 

The above studies not only did not consider strengthening the connection between localization and classification tasks but also lacked in all aspects of network light-weighting, which improves the detection performance to a certain extent but is often accompanied by an increase in network complexity and time cost. Therefore, in view of the above problems and combined with the industrial application scenarios of this article, to fully capture the feature information of small targets, this article proposes a YOLOv8s-DFJA network, which can not only detect stator defects with a high recognition rate and high precision but also meet the requirements of real-time detection.

## 3. Proposed YOLOv8s-DFJA

The YOLOv8s-DFJA network proposed in this article includes the DSFI-HEAD and LEFG modules. The DSFI-HEAD module enhances the network’s positioning and classification performance, and the LEFG module greatly reduces the number of parameters to lighten the entire network.

**Figure 2:**
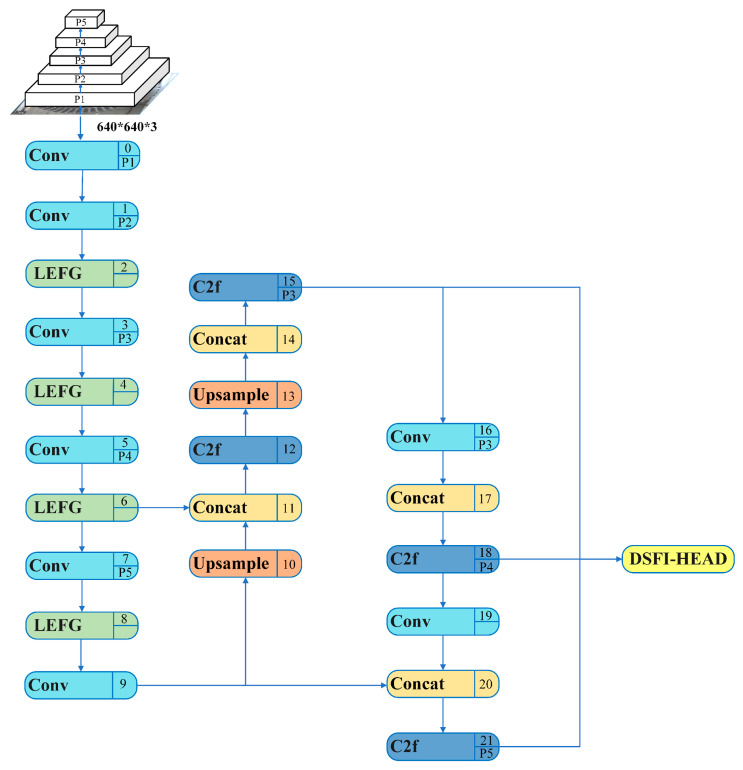
YOLOv8s-DFJA network structure diagram.

The YOLO network series has extremely high similarity [28] regarding network structure. The network mainly has three components: the backbone, neck, and head. The backbone is responsible for extracting features from the input image, which is the basis of target detection in the subsequent network layer. In the YOLOv8 network, the backbone network adopts a structure similar to CSPDarknet, but unlike CSP, YOLOv8 uses a more gradient C2f module. The head network is the decision-making part of the object detection network, responsible for generating the final detection result. In the head part, YOLOv8s replaces the coupling head with the current mainstream decoupling head structure and replaces Anchor-Based with Anchor-Free. The neck network is located between the backbone and head networks for feature fusion and enhancement. YOLOv8s also uses the C2f structure in this part. Based on YOLOv8s, YOLOv8s-DFJA uses the DSFI-HEAD detection head to replace the DETECT detection head in the Head network and the LEFG module to replace the C2f module of the backbone structure. The network structure is shown in Figure 2. Next, this article introduces the structure and function of DSFI-HEAD and LEFG and the effect after fusion.

### 3.1. DSFI-HEAD

**Figure 3:**
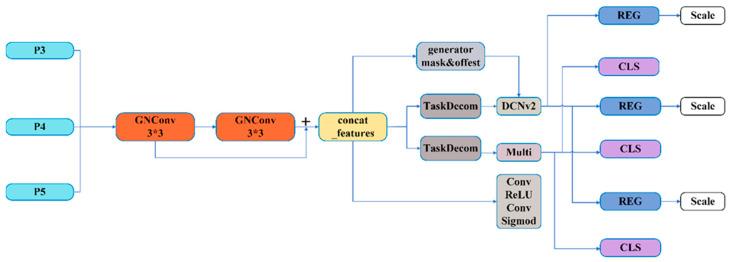
DSFI-HEAD module structure diagram. GNConv has the function of GroupNorm [31] module, which can improve the performance of detection head positioning and classification.

The ROI of this article is the solder joint area of the flat wire motor stator. Although the number of solder joints is large, each solder joint occupies very few pixels. Although the traditional YOLOv8 network improves the detection performance of small targets compared with YOLOv5, it still cannot meet this article’s detection requirements. To improve the detection accuracy of small targets, this article designs a DSFI-HEAD module. The structure of the module is shown in Figure 3. 

To solve the problem of insufficient interaction between classification and localization tasks in traditional object detection networks, this article develops a Dynamic Selection of Feature Interactions Detection Head (DSFI-HEAD) based on the idea of TOOD [29]. The number of output channels of P3, P4, and P5 layers is all adjusted to 512; the feature information of P3, P4, and P5 is input into two information-sharing GNConv. Then, the feature information of the first GNConv module and the feature information after two convolutions are spliced on the channel dimension to form a joint feature. The TAP structure, which is in TOOD, is used in the localization and classification branches after task classification, and the joint features are used in the localization branch to generate mask and offset for DCNv2 [30], which enhances the interaction between tasks. In the classification branch, joint features are used for dynamic feature selection to improve the detection head positioning and classification performance.

Assuming that the feature size of the input to this detector is (B, C, W, H), it is first input to two information-sharing GNConvs. The first GNConv adjusts the number of channels, and the size becomes (B, C//2, W, H). After the second GNConv, the feature size does not change and is still (B, C//2, W, H). The feature information is processed twice by GNConv is spliced on the channel dimension to obtain Concat_features, whose feature size is (B, C, W, H). Figure 3 shows that Concat_features are processed many times, and we introduce them individually:

(1) Concat_features generate offset_and_mask with the size of (B, 3 × 3 × 3, W, H) through the spatial offset convolution layer. Using offset _ dim with the shape of (B, 2 × 3 × 3), the first 18 channels are extracted as offset. The remaining nine channels are used as masks. The extracted mask processed by Sigmod is used to normalize the extracted mask so that offset_and_mask is divided into two parts: offset and mask, that is, classification and regression task decomposition module;
(2) After an adaptive average pooling, the size of Concat_features is changed to (B, C, 1, 1). In the classification and regression task decomposition modules, the feature size is changed to (B, 16, 1, 1) by convolution operation. After ReLU activation processing and convolution again, the size is adjusted to (B, 2, 1, 1), and weight (B, 2) is generated. The weight reshape is (B, 1, 2, 1), which is multiplied by the convolution weight and reshaped as (B, C/2, C). The input feature size is reshaped as (B, C, W * H). After torch.bmm operation, the size becomes (B, C//2, W * H) and is then reshaped as (B, C//2, W, H). After the regression task, the feature information, offset, and mask are input to the DCNv2 layer. The DCNv2 layer is subjected to convolution, normalization, and other operations, and the final output regression feature shape size is (B, C//2, W, H);
(3) Concat_features after Conv, ReLU, and Conv operations of Concat_features are processed by Sigmod to obtain the classification probability. The output features of the classification task and the classification probability are multiplied pixel by pixel to obtain the weighted classification features. Then, through a 1 × 1 convolution layer, the weighted classification features are transformed into the final classification prediction output, and the regression features are transformed into the required output dimension. Because of the shared weight, the detection head cannot detect targets of different sizes. Therefore, after the REG convolution, the feature information is transmitted to the scale layer for feature scaling to realize the function of detecting targets of different sizes. The regression prediction output and the classification prediction output are spliced in the channel dimension to form the final output result.

### 3.2. LEFG

**Figure 4:**
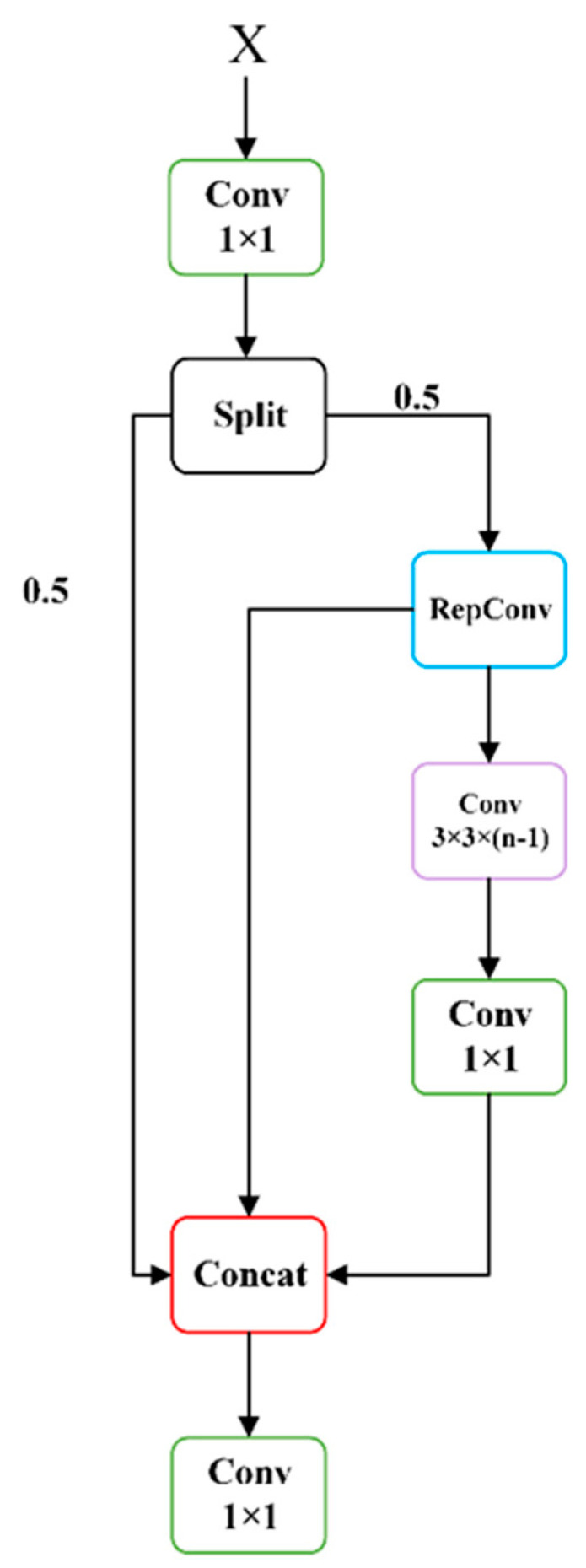
LEFG module structure diagram.

When the mainstream CNN calculates the intermediate feature mapping, there is a lot of parameter redundancy. To solve this problem, this article designs the Lightweight Structure for Enhanced Feature-Extraction and Gradient-Looping (LEFG) module. Using the convolution module with 1 × 1 convolution kernel size to generate partially redundant features instead of the overall redundant features and abandoning the BottleNeck structure used in the traditional YOLO series, the number of parameters is greatly reduced to achieve the purpose of simplifying the computation process and reducing the training cost. To make up for the performance loss caused by discarding the residual structure, the RepCov module is used on the branch of the gradient flow, and feature fusion is performed during reasoning to enhance the ability of feature extraction and gradient flow. The scaling factor can also be adjusted to control the size of the LEFG, improving the speed of YOLOv8s target detection while considering both large and small networks. The structure of the LEFG module is shown in Figure 4.

In this module, n is the number of intermediate convolutional layers, and hidden _ channels are calculated by the factor combination Formula (1) used to determine the number of hidden channels.

(1) $$C_{hidden}=C_{out}\times e$$

This module uses the scaling factor s to scale the number of intermediate channels.

(2) $$C_{mid}=C_{hidden}\times s$$

**Figure 5:**
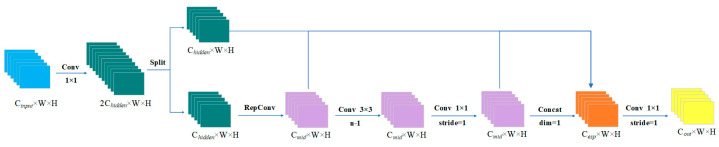
LEFG module dimension variation diagram.

The size change of the feature map is shown in Figure 5:

It is assumed that the feature size input to this module is $\left(C_{input},\;W,\;H\right)$. Firstly, some features are generated by 1 × 1 convolution, and the number of channels is adjusted to 2$C_{hidden}$. Then, the split module is used to perform chunk operation on the channel dimension, and the feature size is divided into two $\left(C_{hidden},\;W,\;H\right)$. The second $\left(C_{hidden},\;W,\;H\right)$ is operated by RepConv, and the feature size becomes $\left(C_{mid},\;W,\;H\right)$. The middle layer contains n-1 convolution layers; each convolution layer is 3 × 3 convolution, and the number of input and output channels are both $C_{mid}$. Therefore, after passing through the middle layer, the feature size is still $\left(C_{mid},\;W,\;H\right)$, and then, the Conv operation with k of 1 × 1 and stride of 1 is performed to obtain the feature size of $\left(C_{mid},\;W,\;H\right)$. At this time, the $\left(C_{hidden},\;W,\;H\right)$ features without any processing, the features obtained by RepConv processing, and the feature information obtained by 1 × 1 Conv operation are spliced on the channel dimension to obtain the feature size $\left(C_{esp},\;W,\;H\right)$.

(3) $$C_{esp}=C_{hidden}+(n+1)C_{mid}$$

Finally, the number of channels is adjusted to $C_{output}$ by 1 × 1 convolution operation.

## 4. Experiment and Discussion

### 4.1. Experimental Condition

The experimental environment of this article involved Ubuntu 20.04, PyTorch 2.1.2, torchvision 0.6.2, and Python 3.9; the batch size is 32, the work number is 32, the initial learning rate is 0.01, the weight attenuation coefficient is 0.0005, input image size is 640 × 640, and the total number of training rounds is 300. The hardware conditions are shown in Table 1 below.

**Figure 6:**
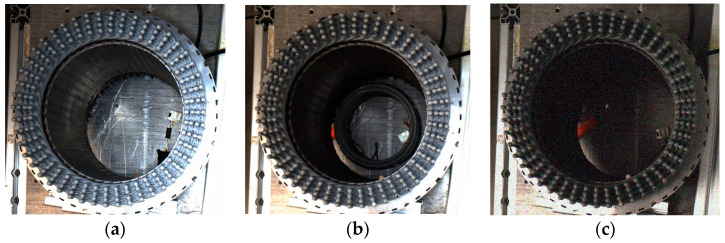
Presentation of datasets: (**a**) original stator image; (**b**) pretzel noise; (**c**) Gaussian blurring.

**Figure 7:**
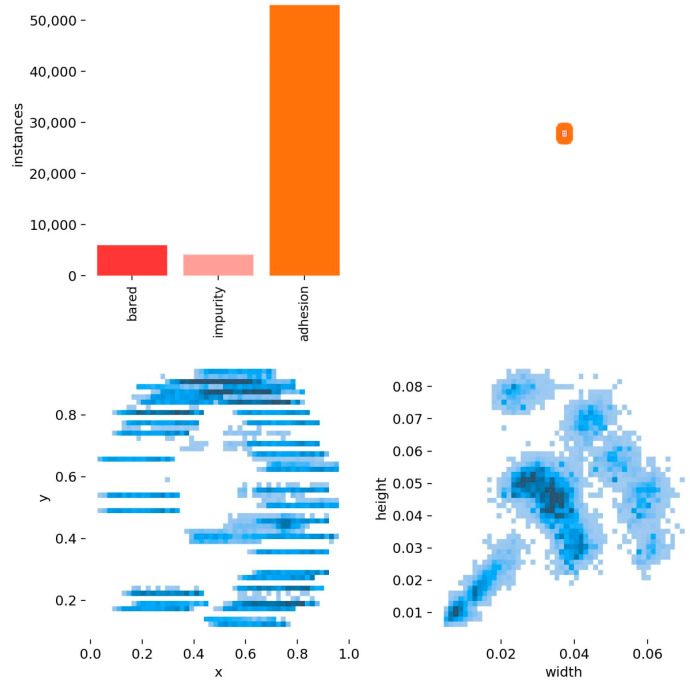
Distribution of dataset information.

The dataset in this article is a small, self-made dataset, and we collected the flat wire motor stator coating defect dataset at FAW TOOLING DIE MANUFACTURING CO., LTD. in Changchun, Jilin Province, China. Using the MV-CS060-10GC camera and the MVL-HF0624M-10MP lens (Hangzhou Haikang Intelligent Technology Co., Hangzhou, china), 611 images were collected on 29 April from 7:30–11:30 and 420 images from 15:30–19:30, and the dataset was expanded to 1705 images using data enhancement techniques (Gaussian noise, pretzel noise, etc.). The dataset has about 72,000 defective targets, and each sample image has at least two defects, so the ratio of the training set, validation set, and test set is about 7:2:1. The dataset is composed of two types of flat wire motor stators provided by FAW TOOLING DIE MANUFACTURING CO., LTD. One kind of flat wire motor stators has four layers of coils with 96 stator grooves in each layer with an outer diameter of 130 mm and an inner diameter of 100 mm; the others have eight layers of coils with 48 stator grooves in each layer, with an outer diameter of 226 mm and an inner diameter of 180 mm; for an example, see Figure 1. The presentation and information distribution of the dataset are shown in Figure 6 and Figure 7.

To objectively evaluate the effectiveness of the network, AP was used as the evaluation index. AP value can reflect the quality of the network. The size of the AP value reflects the strength of the detection ability of the network. The AP value is calculated by precision (P) and recall (R), and the precision-recall (P-R) curve is plotted with P and R as abscissa and ordinate. AP is the area below the P-R curve. The calculation Equation (4) is as follows:

(4) $$AP=\int_{0}^{1}P(R)dR$$

The calculation formulas of P and R are as follows:

(5) $$Precision=\frac{TP}{TP+FP}$$

(6) $$Recall=\frac{TP}{TP+FN}$$

TP stands for true positive, representing the number of positive samples correctly identified by the network; FP stands for false positive, representing the number of negative samples incorrectly identified as positive by the network; FN stands for false negative, representing the number of positive samples that the network failed to detect.

### 4.2. Ablation Experiment of Improved Algorithm

In order to intuitively reflect the differences between networks, parameters such as the size, parameter quantity, and network complexity of each network were compared through experiments. The detailed information is shown in Table 2.

The analysis of Table 2 shows that the parameter quantity of the YOLOv8s network is 10.64 M, the network complexity is 28.6, the inference speed is 72.9 FPS, and the weight file size is 21.4 MB. Based on the YOLOv8 s network, when the DSFI-HEAD module is added alone, the parameter quantity is reduced by 2.16 M, the network complexity is increased by 4.6, and the inference speed is reduced by 11.0 FPS. When only the LFEG module is added, the parameter quantity is reduced by 3.08 M, the network complexity is reduced by 8.1, and the inference speed is increased by 54.4 FPS. When both modules are added, although the network complexity is increased by 1.8, the number of parameters is reduced by 3.00 M, and the inference speed is increased by 9.8 FPS. It is worth noting that the batch size is 1 when calculating FPS. It can be seen that the DSFI-HEAD module and LFEG module designed in this article can reduce the number of parameters.

In order to verify the effectiveness of the proposed YOLOv8s-DFJA network, this study conducted ablation experiments by adding different modules based on the YOLOv8s network. The experiment used the same dataset and training parameters to analyze the influence of the improved method on the detection results. The experimental results are shown in Table 3. It is worth noting that all experiments in this article were trained from scratch and did not use pre-trained weights.

**Figure 8:**
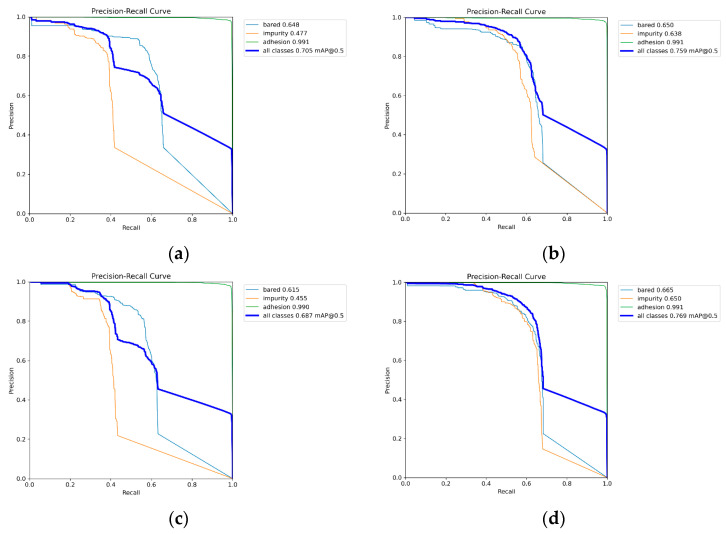
LEFG module dimension variation diagram. PR curves and mAP for each defect category: (**a**) YOLOv8s; (**b**) YOLOv8s-DSFI-HEAD; (**c**) YOLOv8s-LFEG; (**d**) YOLOv8s-DFJA.

Based on the YOLOv8s network and combined with Figure 8 and Table 3, it can be seen that when the DSFI-HEAD module is added alone, the mAP@.5 of the bared category increases by 0.2%, the impurity category increases by 16.1%, and the mAP@.5 of the adhesion category remains unchanged. When only the LFEG module is added, the mAP@.5 of the bared category is reduced by 3.3%, the mAP@.5 of the impurity category is reduced by 2.2%, and the mAP@.5 of the adhesion category is reduced by 0.1%. When both modules are added and effectively combined, the mAP@.5 of the bared category increases by 1.7%, the impurity category increases by 17.3%, and the mAP@.5 of the adhesion category remains unchanged.

It can be confirmed from Table 3 that the DSFI-HEAD detection head proposed in this article is used to replace the original DETECT detection head. Although the network complexity is increased, and more detection time is consumed, the accuracy of detecting the stator defect of the flat wire motor is greatly improved. Using the LFEG module to replace the C2f structure, the parameters such as GFLOPs, parameters, and FPS are all smaller than the original YOLOv8s, which achieves a significant lightweighting of the network structure, but it also brings the disadvantage of insufficient detection accuracy. The organic combination of the DSFI-HEAD detection head and the LFEG module, i.e., YOLOv8s-DFJA uses the LFEG module to replace the traditional C2f module in the backbone part based on YOLOv8s and uses the DSFI-HEAD detection head to replace the Detect detection head in the DETECT layer using the DSFI-HEAD detection head, can further improve the detection accuracy based on the YOLOv8s-DSFI-HEAD network and can also effectively lightweight the network. The detection speed is 9.8 FPS/ss faster than YOLOv8s, while the accuracy is higher than YOLOv8s.

**Figure 9:**
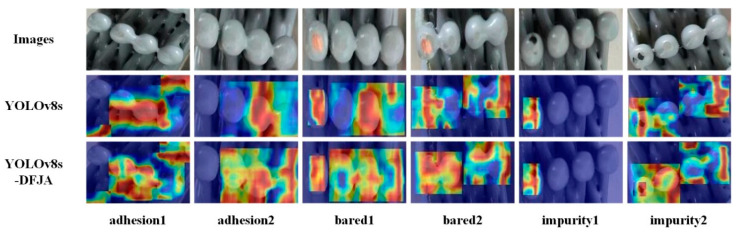
Attention heatmap of the YOLOv8 model and YOLOv8-DFJA model.

The bright and dark regions of the heat map can directly reflect the network’s ability to detect. The heat maps of YOLOv8s-DFJA and YOLOv8s are shown in Figure 9. Compared with YOLOv8s, it can be clearly seen that YOLOv8s-DFJA is more sensitive to bare defects, adhesion defects, and impurity defects and can detect defects more comprehensively. It can be seen that the YOLOv8s-DFJA network proposed in this article has faster inference speed and stronger detection performance.

To further verify that the network proposed in article is robust and generalizable to some extent, we trained it on the public dataset Visdrone2019, and the training results are shown in Table 4.

Table 4 shows that the YOLOv8s-DFJA network proposed in this article still has the highest mAP@.5:.95 on medium-performance devices. It also shows that our network is robust and generalizable to some extent.

In order to more closely match the hardware conditions for deploying deep learning network in real industries, we verified the effectiveness of our network on a medium-performance device. The experimental environment involved Ubuntu 20.04, PyTorch 2.0.0, torchvision 0.15.1, Python 3.8, CUDA 11.8, a batch size of 16, work_num of 32, NVIDIA GeForce RTX2080Ti, Intel(R) Xeon(R) Platinum 8255C CPU @ 2.50 GHz, an initial learning rate of 0.01, a weight decay coefficient of 0.0005, an input image size of 640 × 640, and a total number of training rounds of 300. Training results are shown in Table 5.

Table 5 shows that the YOLOv8s-DFJA network proposed in this article still has the highest mAP@.5 and mAP@.5:.95 on medium-performance devices, which also shows the potential of the network proposed in this paper for practical applications

### 4.3. Comparison with Other Object Detection Algorithms

In this article’s application scenario, the YOLO series networks were used to compare with the proposed YOLOv8s-DFJA network to evaluate its superiority over the proposed YOLOv8s-DFJA network in the YOLO series network. Additionally, for a more comprehensive assessment, comparisons were also made with other mainstream or latest network. The results are shown in Table 6.

Table 6 shows that although the FPS of YOLOv8s-DFJA is not the fastest, it can meet the requirements of the application scenarios in this article. Although the GFLOPs of YOLOv8s-DFJA are not the smallest, the weight files obtained by its training are the smallest, and the number of network parameters is also the least, indicating that the training takes the least time. Combining mAP@.05 and mAP@.05:.95, it can be clearly seen that the YOLOv8s-DFJA network has higher mAP values than other networks. In bared detection, mAP@.5 is 0.6% lower than YOLOv3, and mAP@.5:.95 is 2.4% lower than YOLOv3. According to the above test results, the network has a very high detection accuracy for impurity defects, adhesion of the flat wire motor stator, and copper leakage. In summary, YOLOv8s-DFJA shows the most satisfactory defect detection performance in detecting the coating quality of the stator solder joints of the flat wire motor.

**Figure 10:**
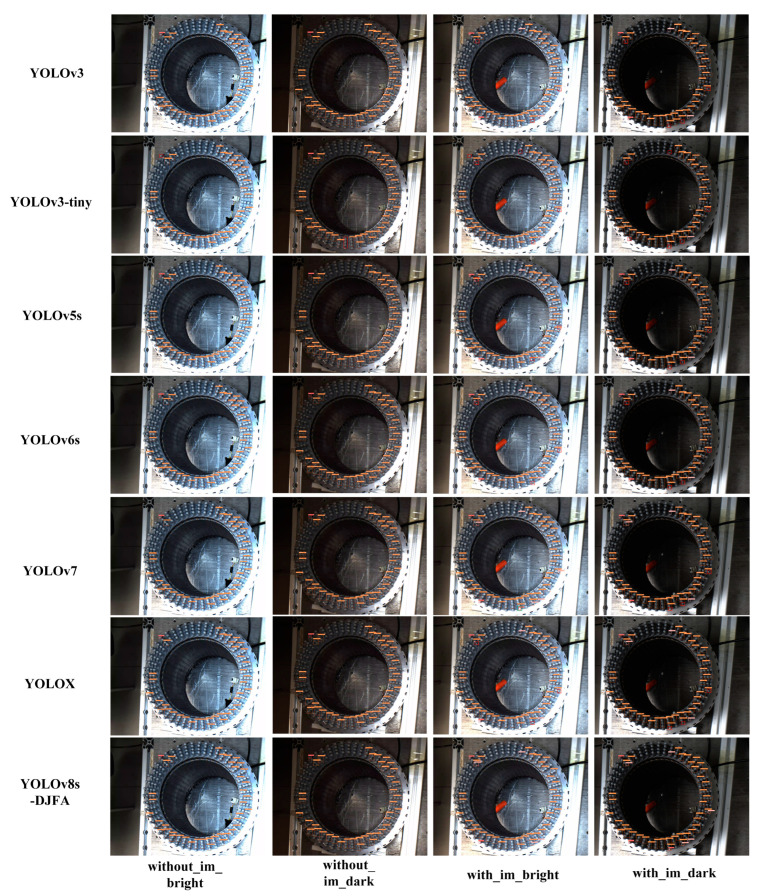
Detection effect diagram of each network. Missing detection targets are marked with thick red frame; false detection targets are marked with thick green frame; im, impurity.

A verification experiment was designed to verify the detection accuracy, as shown in Table 6. The threshold of IoU was set to 0.65, and the threshold of conf was set to 0.25. Four pictures of the same flat wire motor stator, which has eight layers of coils, each equipped with 48 stator slots, were selected in the test set according to the brightness and impurity defects. The detection effect of each network is shown in Figure 10. In Figure 10, the without_im_bright represents a bright sample without impurity defects, the without_im_dark represents a dark sample without impurity defects, the with_im_bright represents a bright sample with impurity defects, and the with_im_dark represents a dark sample with impurity defects. The detection results show that YOLOv8s-DFJA has good detection ability on the test set.

**Figure 11:**
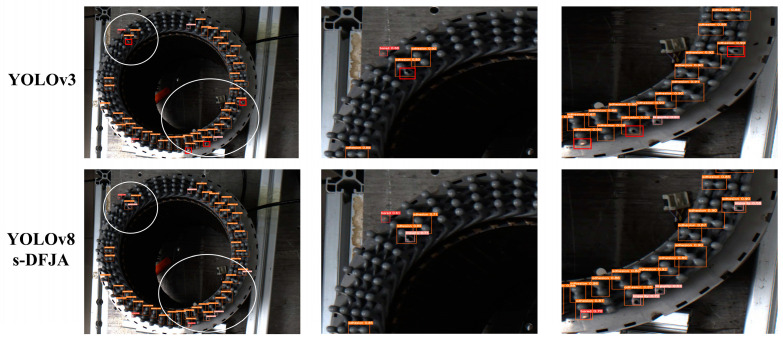
Detection effect of local enlargement.

Because the defect category detected in this article is a small target, the detect effect diagram is locally enlarged to more intuitively reflect the detection effect. We analyzed the data in Table 6 and selected the with_im_bright picture of YOLOv3 and YOLOv8s-DFJA for magnification comparison. The local enlarged image is shown in Figure 11.

By analyzing Figure 11, the following conclusions can be drawn: After 300 epochs of training, although the mAP@.5 and mAP@.5:.95 of the bared category of the YOLOv3 network are higher than the YOLOv8s-DFJA in this article, the effect on the test set is not as good as YOLOv8s-DFJA, and YOLOv3 missed detection. It shows that YOLOv8s-DFJA has high detection accuracy and better robustness and generalization ability than other network.

## 5. Conclusions

Through the experiments of this article, it was found that the YOLOv8s-DFJA network is not only superior to the original YOLOv8s but also superior to many one-stage object detection networks. It can achieve excellent results mainly due to the following two aspects. Firstly, through a feature interaction extractor, the task-related features are extracted and fused from the multi-layer convolutional network to generate a comprehensive and expressive joint feature map. Through deep integration, the synergy between tasks is optimized to improve the accuracy and efficiency of detection performance. Secondly, the LFEG module reduces the parameters and complexity of the network. At the same time, the RepCov module is used on the gradient flow branch to enhance the ability to extract features and gradient flow. The superior performance of YOLOv8s-DFJA and its applicability to real scenes were proven by ablation experiments and other comparative experiments. In the future work, this article will explore how to further enhance the generalization ability of YOLOv8s-DFJA on more areas of industrial defect detection. We hope that Yolov8s-DFJA can be widely used in many kinds of different and complex industrial environments.

## Figures and Tables

**Table 1:** Hardware Conditions.

Name	Configuration
CPU	Inter Xeon(R) Silver4210R CPU@2.40 GHZ*40
GPU	Nvidia GeForce RTX4090*2
Memory	24 G

**Table 2:** Network Parameter Information.

Model	Parameters (M)	GFLOPs	FPS
YOLOv8s	10.64	28.6	72.9
YOLOv8s-DSFI-HEAD	8.48	33.2	61.9
YOLOv8s-LFEG	7.56	20.5	127.3
YOLOv8s-DFJA	7.64	30.4	82.7

**Table 3:** Ablation Experiment Results of YOLOv8 Network.

Method	Class	P (%)	R (%)	mAP@.5 (%)
YOLOv8s	All	84.9	65.1	70.5
YOLOv8s-DSFI-HEAD		87.0	70.8	75.9
YOLOv8s-LFEG		86.1	64.2	68.7
YOLOv8s-DFJA		86	73.2	76.9
YOLOv8s	Bared	84.4	56.4	64.8
YOLOv8s-DSFI-HEAD		85.2	57.5	65
YOLOv8s-LFEG		79.7	56.4	61.5
YOLOv8s-DFJA		83.7	58.9	66.5
YOLOv8s	Impurity	72.8	39.4	47.7
YOLOv8s-DSFI-HEAD		78.3	55.7	63.8
YOLOv8s-LFEG		81.1	36.9	45.5
YOLOv8s-DFJA		77.4	61.6	65
YOLOv8s	Adhesion	97.5	99.3	99.1
YOLOv8s-DSFI-HEAD		97.4	99.3	99.1
YOLOv8s-LFEG		97.4	99.2	99
YOLOv8s-DFJA		98	99.3	99.1

**Table 4:** Ablation Experiment Results of YOLOv8 Network on Visdrone2019.

Model	All		Parameters (M)	GFLOPs	Weight_Size	FPS
	mAP@.5	mAP@.5:.95				
YOLOv8s	0.396	0.236	10.61	28.5	22.5	49.3
YOLOv8s-DSFI-HEAD	0.409	0.248	8.48	33.0	18.0	46.3
YOLOv8s-LFEG	0.382	0.226	7.53	20.3	16.3	67.2
YOLOv8s-DFJA	0.414	0.249	7.63	30.4	16.4	58.8

**Table 5:** Ablation Experiment Results of YOLOv8 Network with NVIDIA GeForce RTX2080Ti.

Model	All		Parameters (M)	GFLOPs	Weight_Size	FPS
	mAP@.5	mAP@.5:.95				
YOLOv8s	0.685	0.442	10.61	28.4	21.4	41.2
YOLOv8s-DSFI-HEAD	0.746	0.459	8.47	33.0	17.1	44.2
YOLOv8s-LFEG	0.673	0.422	7.51	20.9	15.5	69.4
YOLOv8s-DFJA	0.751	0.46	7.63	30.4	15.6	50.8

**Table 6:** Comparative Experimental Results of Other Object Detection Algorithm.

Model	All		Bared		Impurity		Adhesion		Parameters (M)	GFLOPs	Weight_Size	FPS
	mAP@.5	mAP@.5:.95	mAP@.5	mAP@.5:.95	mAP@.5	mAP@.5:.95	mAP@.5	mAP@.5:.95				
YOLOv3	0.716	0.448	0.671	0.309	0.488	0.245	0.989	0.791	98.86	282.2	198.2	102.3
YOLOv3-tiny	0.545	0.362	0.383	0.169	0.262	0.111	0.99	0.804	11.57	18.9	23.3	337.3
YOLOv5s	0.682	0.435	0.649	0.291	0.406	0.197	0.991	0.816	8.69	23.8	18.5	134.6
YOLOv6s	0.682	0.439	0.624	0.292	0.435	0.209	0.991	0.817	15.54	44.0	32.9	140.3
YOLOv7	0.693	0.381	0.458	0.151	0.634	0.243	0.987	0.748	34.80	103.2	74.8	64.5
YOLOX-s	0.755	0.453	0.640	0.281	0.636	0.281	0.991	0.796	8.94	26.76	71.8	61.3
YOLOv9s	0.686	0.424	0.629	0.274	0.441	0.207	0.99	0.79	6.84	26.7	15.3	44.8
SSD	0.664	0.403	0.588	0.246	0.413	0.179	0.99	0.782	23.5	230.6	96.1	59.8
YOLOv8s-DFJA	0.769	0.463	0.665	0.285	0.650	0.285	0.991	0.819	7.64	30.4	15.7	82.7

## Data Availability

VisDrone2019 Dataset: The VisDrone2019 dataset used in this study is publicly available and can be accessed from the official GitHub repository: https://github.com/VisDrone/VisDrone-Dataset (accessed on 11 August 2024). Our dataset: We can’t make the dataset public because the company’s experiments on it are not yet complete and are still in the trial phase.
